# Animal models and SARS-CoV-2-induced pulmonary and neurological injuries

**DOI:** 10.1590/0074-02760220239

**Published:** 2023-01-20

**Authors:** Marcelo Alves Pinto, Alexandre dos Santos da Silva, Daniela Del Rosario Flores Rodrigues, Rodrigo Müller, Gentil Arthur Lins Bentes Mendonça de Vasconcelos, Patrícia Cristina da Costa Neves, Jaqueline Mendes de Oliveira, Renato Sergio Marchevsky

**Affiliations:** 1 Fundação Oswaldo Cruz-Fiocruz, Instituto Oswaldo Cruz, Laboratório de Desenvolvimento Tecnológico em Virologia, Rio de Janeiro, RJ, Brasil; 2 Fundação Oswaldo Cruz-Fiocruz, Instituto de Tecnologia em Imunobiológicos, Laboratório de Experimentação Animal, Rio de Janeiro, RJ, Brasil; 3 Fundação Oswaldo Cruz-Fiocruz, Instituto de Tecnologia em Imunobiológicos, Vice-Diretoria de Desenvolvimento Tecnológico, Laboratório de Tecnologia Imunológica, Rio de Janeiro, RJ, Brasil; 4 Fundação Oswaldo Cruz-Fiocruz, Instituto de Tecnologia em Imunobiológicos, Laboratório de Neurovirulência, Rio de Janeiro, RJ, Brasil

**Keywords:** SARS-CoV-2, animal models, neurological injury, respiratory injury, experimental pathology

## Abstract

Laboratory animals are essential mainly for experiments aiming to study pathogenesis and evaluate antivirals and vaccines against emerging human infectious diseases. Preclinical studies of coronavirus disease 19 (COVID-19) pathogenesis have used several animal species as models: transgenic human ACE2 mice (K18 mice), inbred BALB/c or C57BL/6N mice, ferrets, minks, domestic cats and dogs, hamsters, and macaques. However, the choice of an animal model relies on several limitations. Besides the host susceptibility, the researcher’s experience with animal model management and the correct interpretation of clinical and laboratory records are crucial to succeed in preclinical translational research. Here, we summarise pathological and clinical findings correlated with virological data and immunological changes observed from severe acute respiratory syndrome coronavirus 2 (SARS-CoV-2) experimental infections using different well-established SARS-CoV-2 animal model species. This essay aims to critically evaluate the current state of animal model translation to clinical data, as described in the human SARS-CoV-2 infection.

Recently, there has been a consensus about the contribution of the animal model approach to studying pathogenesis and evaluating antivirals and vaccines against the severe acute respiratory syndrome coronavirus 2 (SARS-CoV-2). In February 2020, the World Health Organization (WHO) assembled an international panel to developing animal models for coronavirus disease 2019 (COVID-19) to accelerate the testing of vaccines and therapeutic drugs.^1^ This review summarises the current state of COVID-19 pre-clinical studies using well-established animal models to study pathogenesis and evaluate antivirals and vaccines, highlighting the SARS-CoV-2-induced pulmonary and neurological injuries. This essay contemplates a critical evaluation of pre-clinical studies and their contribution to the clinical management of COVID-19, as described in the recent medical literature.

SARS-CoV-2 infection in human beings

The clinical evolution of human SARS-CoV-2 infection ranges from asymptomatic to severe respiratory failure requiring mechanical ventilation.^2^ Approximately 80% of cases have a benign evolution, being clinically considered as “mild COVID-19” developing with coughing, anosmia, ageusia, mild gastrointestinal symptoms, shortness of breath, and a low-grade fever. In a “moderate” clinical evolution, pneumonia occurs in approximately 15% of the cases; from the tenth day, symptoms begin to worsen, with dyspnoea, and a consequent decrease in oxygen saturation. Such a severe evolution is suggestive of infection in the lower respiratory tract, showing detectable lung inflammatory activity and prothrombotic components.^3^ A “severe COVID-19” is more likely to occur in patients who have at least one comorbidity, e.g., obesity, diabetes, metabolic and cardiovascular diseases.^4^ Hypertension (56.6%) and diabetes (33.8%) are the most prevalent comorbidities, along with severe COVID-19 requiring hospitalisation. The SARS-CoV-2 tropism, and the interaction of spike (S) glycoproteins with the renin-angiotensin-aldosterone system, through ACE2 receptor, may enhance the cardiac inflammatory injury.^5^

The severe disease, which occurs in approximately 5% of the cases, is characterised by pneumonia with worsening respiratory condition, hypoxemia, fever, inflammatory hyperactivity, and bilateral pulmonary ground-glass opacities depicted by computed tomography (CT) imaging.^2^ The hyperactivation of the immune system may induce a process known as immunothrombosis, in which activated neutrophils and monocytes interact with platelets and the coagulation cascade, leading to intravascular clot formation in small and larger vessels. Microthrombus contribute to acute respiratory distress syndrome and other systemic dysfunctions.^6^

The SARS-CoV-2-induced endothelial dysfunction^7^ ^,^ ^8^ leads to tissue factor (TF) exposure with consequent activation of the coagulation cascade, thus generating a state of hypercoagulability, thromboembolic events, and increased risk of bleeding by disseminated intravascular coagulation (DIC).^9^ Therefore, systemic viral inflammation contributes to the multiple organ dysfunction syndrome.^10^ Additionally, SARS-CoV-2 RNA and antigen load in blood has been associated with worse disease outcomes and death with systemic viral dissemination.^11^ There have been frequently observed atypical large basophilic structures in the liver sinusoidal endothelium and increased immunoblast-like cells in lymph nodes.^12^ The SARS-CoV-2-induced lung injury of deceased patients was summarised by Fiorella and colleagues as follows: lung consolidation, interstitial congestion, haemorrhage, pleural effusions and adhesions, bronchopneumonia, mucous plugs, macro and microvascular thromboembolism, diffuse alveolar damage (DAD), hyaline membrane deposition in alveolar surface, necrosis, prominent desquamating pneumocyte II hyperplasia, with focal multinucleated and bizarre cell forms.^13^

SARS-COV-2 infection-inducing neural injuries in human

The neuroinvasion of viruses in the human brain has been observed, despite the external multilayer blood-brain barrier and effective immune responses.^14^ Theoretically speaking, viruses can enter the central nervous system (CNS) through the hematogenous or neuronal retrograde routes.^15^ The local immune activation may result in direct immune-mediated injury.^14^ Viruses can infect central and peripheric nervous systems either by direct infection of nerve endings or by infecting cells of the circulatory system that, ultimately, carry the infection through the blood-brain barrier into the CNS.^16^ Endothelial cells of the blood-brain barrier or epithelial cells in the choroid plexus and leukocytes may play a role as vectors for dissemination within the CNS when the activated microglia,^17^ the residing mononuclear phagocytes of the brain, act as the first line of defence and start the neuroinflammatory events.^14^

Thus, the local inflammatory responses may cause neuronal damage. However, whether or how SARS-CoV-2 reaches the CNS is unclear since only a few patients with COVID-19 have detectable SARS-CoV-2 RNA (RNAemia) in the blood.^11^ Considering the structural similarities between SARS-CoV-2 and SARS-CoV and their target receptor, AC2,^14^ it is possible to infer from evidence of SARS-CoV neurovirulence and neurotropism provided by several studies.^18^ So far, the invasion of peripheral nerve terminals, especially the olfactory and vagus nerve, are hypothetically the routes that lead to CNS infection.^19^

To date, pieces of evidence for SARS-CoV-2 neurotropism are scarce. Besides the olfactory dysfunction that affects many SARS-CoV-2 infected people, neurological disorders such as encephalopathy, encephalitis, meningoencephalitis, acute myelitis, and Guillain-Barré syndrome have been described in COVID-19 patients.^14^ Additionally, there is cumulative evidence for a link between the development of acute cerebrovascular diseases, e.g., stroke and the severity of COVID-19 disease, particularly in patients with cardiovascular comorbidity.^5^

Animal models as a translational approach to studying COVID-19 pathogenesis

Studies aiming to assess the susceptibility of animal species to SARS-CoV-2 rely on the implied affinity of their angiotensin-converting enzyme 2 (ACE2) and its capability to function as receptor-binding domain sites for the viral spike protein. Old World macaques present high susceptibility, pangolins and cats present medium to high, whereas rats and mice present low or very-low susceptibility to SARS-CoV-2 infection.^20^ Macaques are susceptible to several human viruses, which replicate in the same target cells, whose infections result in similar pathologies and cross-reacting immune responses.^21^ Experimental studies demonstrated rhesus macaques (*Macaca mulatta*) as the most susceptible species to SARS-CoV-2 infection, followed by cynomolgus (*Macaca fascicularis*) and, in a lesser likelihood, common marmosets (*Callithrix jacchus*).^22^

SARS-COV-2 infection in transgenic human ACE2 mice and inbred mice models

Experimental studies using K18-hACE2 mice show that SARS-CoV-2 infection ranges from asymptomatic to severe respiratory disease. Ordinarily, SARS-CoV-2-infected mice exhibit mild symptoms followed by recovery; severe lung and brain injuries have rarely been observed.^23^ Despite the mild clinical signs, with weight loss not above 5% and absence of respiratory symptoms, approximately 60% of the mice survive after the fifth days post-infection (dpi), thus suggesting that mortality probably occurs due to SARS-CoV-2 neuroinvasion and local inflammation.^24^ Controversially, Zheng and colleagues (2021) described extensive and progressive lung damage; in some mice, SARS-CoV-2 was also detected in the brain. The histological analysis of the lungs revealed evidence of diffuse alveolar damage with progressive alveolar/interstitial lesions characterised by oedema, inflammation, and focal cytomegaly in some alveolar lining cells. The alveolar septum showed accumulation of immune effector cells, including granulocytes and macrophages, cell deaths, haemorrhage, hyaline membranes lining the alveolar surface, occasional vascular thrombi, and rare syncytia. There was progressive and diffuse alveolar damage between the fourth and sixth dpi. Other findings included alveolar septal thickening with increased infiltrating inflammatory cells, dying cells with pyknotic to karyorrhectic nuclei, and proliferative alveolar epithelium with mitotic cells.^24^

Of note, there was evidence of thrombosis and vasculitis in some (but not all) mice with severe interstitial pneumonia. Animals showed clinical signals of anosmia early after infection, probably due to the presence of ACE2 in sustentacular cells in the olfactory epithelium. At the sixth dpi, an extensive antigen staining (N protein) was observed in the olfactory bulb, cerebral cortex, caudate/putamen, thalamus, hypothalamus, and ventral striatum. These findings suggest that the sustentacular cells may be a primary site of infection and still contribute to anosmia.^24^

In another study, after intratracheal instillation of the S1 subunit of SARS-CoV-2 spike protein (S1SP), the K18-hACE2 transgenic mice reproduced signs of COVID-19-associated lung injury. At the third dpi, animals had a 10% decline in body weight, increased white blood cell count and cytokine storm in bronchoalveolar lavage fluid and serum, associated with histological evidence of lung injury.^25^ Other authors described mild-to-moderate pneumonia associated with hypothermia and viral neural dissemination, independent of the inoculation doses.

Recent studies using two lethal mouse-adapted SARS-CoV-2 variants (BMA8 and C57MA14) induced high viral replication titters in the upper and lower respiratory tract pulmonary, cytokine storm, cellular tropism, lymphopenia, and neutrophilia in young BALB/c or C57BL/6N models.^26^ In the K18-hACE2 mice model, severe neuroinvasion was observed at the fourth dpi, initially restricted to the olfactory bulb, suggesting axonal transport via the olfactory neuroepithelium as the earliest portal of entry. In the absence of viremia, the authors assumed that neuroinvasion occurred independently of transport across the blood-brain barrier.^27^

In another experiment using K18-hACE2 mice, 50% of the SARS-CoV-2 infected animals exhibited CNS infection with viral replication in neurons accompanied by increased expression of inflammatory mediators’ transcripts associated with microgliosis and neuroinflammation - primarily, monocytes/macrophages infiltrate. These findings contribute to confirming the ability of SARS-CoV-2 to infect neurons and emphasise the use of the K18-hACE2 model to study neuropathological aspects related to SARS-CoV-2-induced neurologic disease.^17^

**SARS-COV-2 infection in ferrets and minks (*Mustelidae* family)**

Ferrets have been used for evaluating the pathogenicity, transmissibility, and tropism of influenza viruses and several other human respiratory viruses,^28^ including the respiratory syncytial virus,^29^ parainfluenza^30^ and SARS-CoV infections.^31^ Although SARS-Cov-2 infection of mustelids does not cause severe pulmonary injury, transmission has been confirmed experimentally, either by inoculation via the intratracheal route or from an infected ferret to a naive one.^32^ The SARS-CoV-2 infected ferrets presented fever (from 38.1 to 40.3ºC), loss of appetite and weight, occasional coughs, and reduced mobility in the cage. The main histopathological features were severe lymphoplasmacytic perivasculitis and vasculitis in the lungs, mild peribronchitis, and inflammatory cell infiltration in the alveolar septa and lumen. Viral replication was evidenced by the detection of viral antigens in the nasal turbinate, tonsils, and soft palate, indicating virus replication in the upper respiratory tract, with virus shedding in nasal washes, urine, and faeces of the SARS-CoV-2 infected ferrets. However, the SARS-CoV-2 infected ferrets showed only mild clinical respiratory signals, despite the acute bronchiolitis observed in some animals.^32^

SARS-CoV-2 is a zoonotic virus, which can spill over from another animal species to infect humans and vice-versa; for instance, humans can infect domestic cats and breeding animals in zoos. Munnink and colleagues reported the spillover of SARS-CoV-2 on mink farms in the Netherlands, as evidenced by transmission between humans and minks and back to humans.^33^ The main symptom of SARS-CoV-2 infection in minks was respiratory discharge.^34^ Human-to-mink and mink-to-human transmission was also reported in a breeding farm in Poland, with a stock of 5,850 minks. Notwithstanding, there is no evidence of SARS-CoV-2 systemic infection nor neurotropism in SARS-CoV-2-infected mustelids.^35^

**SARS-COV-2 infection in domestic felines (*Felis catus*) and wild animals**

The natural susceptibility of domestic cats and wild felines to SARS-CoV-2 has been investigated since the early COVID-19 global pandemic.^36^ ^,^ ^37^ ^,^ ^38^ ^,^ ^39^ ^,^ ^40^ It is assumed that ACE2 receptors from domestic cats, tigers and Lions could efficiently interact with the receptor-binding domain RBD in the SARS-CoV-2 spike protein.^41^ Domestic cats can be naturally infected by close contact with SARS-CoV-2-infected patients; human-to-pet or pet-to-human transmission occasionally occurs via respiratory secretions.^42^ ^,^ ^43^ The experimental SARS-CoV-2 infection induces a non-lethal respiratory disease in felines, like asymptomatic or mild human SARS-CoV-2 infections in humans.^38^

The lung parenchyma of cats occasionally shows minimal to mild inflammation, mainly composed of lymphocytes and macrophages, a few plasmocytes, and neutrophils. The infection showed moderate hyperplasia of the epithelium, with a layering of nuclei and multifocal herniation of epithelium below the smooth muscle layer. Lymphocytes occasionally migrate into the epithelium. Bronchial lumina infrequently contains sloughed epithelial cells or mucus, which occlude less than 25% of the lumen. Mild to moderate hyperplasia of the bronchus-associated lymphoid tissue, composed of T and B lymphocytes, has been also reported.^40^

In March 2020, four Siberian tigers (*Panthera tigris*) and three African lions (*Panthera leo*) at the Bronx Zoo, NY, developed mild respiratory signs (dry cough and wheezing), with SARS-CoV-2 RNA detected in their respiratory secretions and or faeces; three had the virus isolated, and one showed cellular damage with viral RNA colocalised. The epidemiologic and genomic data suggested a human-to-tiger transmission. The infected animals shed infectious virus particles in respiratory secretions and faeces.^44^ Wild ruminants such as white-tailed deer (*Odocoileus virginianus*) show susceptibility after experimental intranasal inoculation becoming subclinically infected and shedding infectious SARS-CoV-2 in nasal secretions and faeces.^45^

**SARS-COV-2 infection in Golden Syrian and Roborovski hamsters (*Cricetinae* family)**

Members of the *Cricetinae* family express ACE2 in their cell membrane surfaces in a high homology with the human enzyme, thus providing host cell invasion and replication. Recently, Tomris and colleagues (2022) demonstrated that the SARS-CoV-2 experimental infection in golden (Syrian) hamsters is restricted to sites containing both ACE2 (predominantly observed in the bronchioles and alveoli) and the transmembrane serine protease 2 (TMPRSS2), expressed in the primary and secondary bronchi.^46^ Golden Syrian hamster (*Mesocricetus auratus*), an outbred available animal, has been widely used in COVID-19 preclinical studies.

The SARS-CoV-2 intranasal infection of Syrian hamsters results in a moderate to severe respiratory disease, affecting the upper airways and lung parenchyma. Mild to moderate mixed- inflammation in the airway lumen and multifocal interstitial mononuclear cell inflammation have been observed, at early and late time points, without deaths.^26^ ^,^ ^47^ In contrast, Roborovski hamsters (*Phodopus roborovskii*), similar to transgenic human ACE2 hamsters, develop severe respiratory and systemic disease and death, presenting clinical respiratory signals like the severe human COVID-19: snuffling, laboured breathing, anosmia, dyspnoea, cough, hunched posture, progressive weight loss, ruffled fur, fever, and shaking chills, with right-predominated interstitial pneumonia and slight damage to the brain and liver.^48^ ^,^ ^49^

It has been also demonstrated that SARS-CoV-2-infected hamsters exhibit pulmonary upregulation of inflammatory mediators that persist upon virus clearance and increased apoptosis and ferroptosis in their epithelial cells.^50^ Mainly, aged hamsters (above 36 weeks old) show prolonged prothrombin time by the consumption of coagulation factors leading to prothrombin time prolongation and increased intravascular coagulation in the lungs, acute kidney injury in the proximal urinary tract and expansion of the mesangial matrix, and severe acute respiratory syndrome.^51^

The hamster’s olfactory neuroepithelium is the primary site of SARS-CoV-2 neural replication. Consequently, the inflammation of the olfactory sensory neurons, support cells, and immune cells causes acute anosmia and ageusia. The local inflammation lasts as long as the virus remains in the olfactory epithelium and olfactory bulb, similar to the long-term persistence of COVID-19-associated anosmia in humans, with virus transcripts detected in SARS-CoV-2-infected cells.^52^

Hamsters infected via nasal instillation of the SARS-CoV-2 S protein showed microglia activation and inflammatory injury in the olfactory bulb, with a significant correlation between the level of anosmia and the histological damage score within the olfactory epithelium. The thickness of the olfactory bulb epithelium, due to the local inflammation upon infection, seems to affect the food-searching behaviour and, consequently, cause body weight loss.^53^ ^,^ ^54^ Other brain regions were not affected, thus suggesting a selective neural vulnerability.^55^

**SARS-CoV-2 infection in non-human primates (*Macaca mulatta*, *Macaca fascicularis*, and *Callithrix jacchus*)**

Identifying the direct cellular targets of SARS-CoV-2 infection within human or non-human primate hosts is central to understanding COVID-19 pathogenesis. Metanalysis of single-cell-RNA-sequencing datasets have indicated potential SARS-CoV-2 targets in subsets of ciliated, goblet, and secretory cells from oropharyngeal, nasal, and upper airway tissues.^52^ It is known that AC2 and TMPRSS2 targets are co-expressed within the lung parenchyma type II pneumocytes, ileal absorptive enterocytes, and nasal goblet secretory cells. However, ACE2 protein expression within the respiratory tract of macaques is controversial (22). Human- and rhesus-ACE2 amino acid sequences share a homology of 91%,^56^ which can be why rhesus monkey ACE2 receptor support an efficient virus entry.^57^ In a study using rhesus monkeys (*Macaca mulatta*) inoculated with SARS-CoV-2 via the intratracheal route, animals presented mild disease and weight loss but no fever. From the first to the 14th dpi, viral RNA was detected in the oropharyngeal and anal swab samples.^58^

Another study demonstrated clinical signs, including weight loss and asthenia. In contrast with young monkeys, older animals showed a higher virus replication in the lung, and in nasopharyngeal and anal swabs. Besides, they developed severe typical interstitial pneumonia with inflammation and oedema; specific antibody conversion occurred 14 days post-infection.^59^

SARS-CoV-2 infected rhesus monkeys present a mild clinical disease, with decreased appetite and responsiveness, even though they have evidence of pneumonia, with multifocal lung inflammation. High levels of viral RNA can be detected in the nasal mucosa, pharynx, trachea, and lung tissues but not in the gastrointestinal tract, liver, and kidney. Animals rechallenged after 35 dpi were protected against reinfection, showing lower RNA titres in the bronchoalveolar lavage than in nasal swabs, faster immune responses, and mild or no clinical disease.^60^

Regarding the cynomolgus (*Macaca fascicularis*) model, experimental infection with SARS-CoV-2 induces *in foci* or diffuse alveolar damage in type I and II pneumocytes and ciliated epithelial cells of the nasal, bronchial, and bronchiolar mucosae; viral excretion is mainly detectable from the nose and throat. Seroconversion against the SARS-CoV-2 S1 domain and nucleocapsid proteins, with viral replication on the respiratory tract, can be detected about the 14th dpi.^61^

In general, macaques experimentally infected with SARS-CoV-2 may demonstrate a mild infection, with thickened alveolar septa and monocyte infiltration. In contrast, when severely infected, macaques present acute diffuse alveolar damage, with extensively broadened alveolar septa, increased monocyte infiltration, visible exudation of protein-rich oedema fluid in the alveolar cavities, damaged alveolar fibres, and local fusion of thick septa. SARS-CoV-2-specific viral antigens can be detected in the lung of both mildly and severely infected animals.^62^ Either rhesus or cynomolgus macaques did not show significant weight loss or changes in body temperature throughout the experiment.

The absence of clinical signs contrasts with the CT images from the lungs, collected about 18 dpi from rhesus and cynomolgus macaques; pulmonary damage affects less than 25% of the lung. Ground glass opacity can also be observed, with the abnormal structure of the lungs and peripheral consolidation. Pulmonary abnormalities can occur in two-thirds of the middle and lower lung lobes of cynomolgus, in a random pattern, in the upper, middle, and lower lobes of rhesus macaques; microthrombus and emboli have not been identified.^19^

Neuroinvasion of SARS-CoV-2 was recently elucidated in rhesus monkeys. SARS-CoV-2 invades the CNS, primarily via the olfactory bulb and rapidly spreads to the hippocampus, thalamus, and medulla oblongata, thus inducing inflammation in targeting neurons, microglia, and astrocytes, as confirmed by *in vitro* infection using neuro-derived SK-N-SH, glial-derived U251, and brain microvascular endothelial cells.^19^ Despite phylogenetic similarities, there is no evidence in the present scientific literature of the circulation of SARS-CoV-2 in neotropical monkeys.^63^

In conclusion

Several animal species have been used as models to study SARS-CoV-2 pathogenesis and the efficacy of treatment and prevention. The best choice relies upon the SARS-CoV-2-inducing damages is expected the host model will exhibit. The hACE-2 K18 mice are considered the best model to assess the neurotropic effect, ageusia and anosmia treatment. Young hamsters and macaques have been used for assessing encephalitis and mild respiratory disease, whereas adult animals are preferred to evaluate respiratory and systemic moderate disease. Aged hamsters and macaques are models to assess the severe respiratory and systemic disease. Ferrets, minks, and felines reproduce the natural host transmission and the upper respiratory disease. The main limitation in Brazil and other Latin American countries is the high costs of Animal Biosafety Level 3 (ABSL-3) animal facilities, which universities and research institutes support. Also, the researcher’s experience with animal model management and the correct interpretation of clinical and laboratory records are crucial to succeed in preclinical translational research.
